# Evaluation of Total Volatile Basic Nitrogen, Formaldehyde, and Formic Acid as Markers to Define the Acceptability of Farmed Sea Bass and Sea Bream Stored Under Vacuum (VP) or in Modified-Atmosphere Packaging (MAP) at 4 ± 2 °C

**DOI:** 10.3390/microorganisms13122774

**Published:** 2025-12-05

**Authors:** Michela Pellegrini, Debbie Andyanto, Lucilla Iacumin, Giuseppe Comi

**Affiliations:** Department of Agricultural, Food, Environmental and Animal Sciences, University of Udine, Via Sondrio 2/a, 33100 Udine, Italy; michela.pellegrini@uniud.it (M.P.); debbie.andyanto@uniud.it (D.A.); lucilla.iacumin@uniud.it (L.I.)

**Keywords:** sea bass, sea bream, microbial and physico-chemical loads, TVB-N, acceptability

## Abstract

The aim of this work was to propose an objective parameter to define the acceptability of fresh sea bass and sea bream among concentrations of formaldehyde, formic acid, and TVB-N. As indicated, TVB-N appeared to be the most appropriate index. The formaldehyde value cannot be used because it increased until day 6 and then decreased because it was transformed into formic acid. The decrease was observed at all of the times tested. Nevertheless, formic acid also cannot be considered as a valid parameter because in both of the tested fish, it reached values of less than 7.2 mg/kg at 15 days of storage, even though the sensorial analysis indicated the loss of acceptability. In addition, this value is 4–7 times lower than the concentration present in other fresh fish. Consequently, TVB-N represents the only parameter of interest for defining acceptability for both fish species and it can be accepted as the freshness index. Considering the results of the microbial, physico-chemical, and sensory analysis, a level of TVB-N less or equal to 35 mg N/100 g of product was observed up to 12 days of storage. Then, at 15 days, TVB-N reached values over 40 mg N/100 g and both the fish were no longer acceptable, as demonstrated by sensory analysis. For this reason, this value can also be proposed as the limit of freshness for sea bass and sea bream, stored either in VP or in MAP at 4 ± 2 °C. Considering the microbial, physico-chemical, and sensorial analysis of both fish species, a shelf-life limit of 12 days was proposed.

## 1. Introduction

European sea bass (*Dicentrarchus labrax*) and gilthead sea bream (*Sparus aurata*) represents the most important marine fish species farmed in Europe, with a particularly high relevance in Mediterranean countries, due to their high consumer acceptance, favorable nutritional profiles, and economic relevance [1,2,3,4,5]. Consequently, extensivefishrch has focused on the influence of rearing systems, feeding regimes, and post-harvest handling on product quality, given that these parameters significantly affect the composition, texture, and hygienic characteristics of the fish flesh [6,7]. Despite their economic importance, both sea bass and sea bream are highly perishable commodities. Deterioration begins immediately after death, mainly due to the combined action of endogenous enzymes and microbial metabolism, which strongly influence the rate of spoilage and shelf life [8,9]. The loss of freshness is a progressive and multifactorial process whose kinetics depend on handling, slaughtering, and storage conditions, including temperature and atmospheric compositions [9,10,11]. Initially, autolytic reactions dominate, triggered by endogenous proteases and nucleases that act on muscle proteins and non-protein nitrogen (NPN) compounds, leading to tissue softening and the release of substrates suitable for microbial growth [12,13,14,15]. During post-mortem metabolism, ATP is sequentially degraded into inosine-5′-monophosphate (IMP), which is associated with pleasant, fresh flavor, and then into inosine and hypoxanthine, compounds responsible for stale odors and bitterness [13,14,15]. As autolysis progresses, different microorganisms become the main drivers of spoilage. *Pseudomonas*, *Shewanella*, *Photobacterium*, *Aeromonas*, and *Brochothrix thermosphacta* dominate during storage, reaching levels exceeding 8 log CFU g^−1^ or cm^−2^ [16,17,18,19,20,21] and are responsible for the development of off-odors and off-flavors [8,22,23]. Spoilage patterns are highly dependent on temperature and packaging atmosphere; under aerobic conditions, *Pseudomonas* spp. dominate, whereas under modified-atmosphere packaging (MAP), *Pseudomonas* growth is suppressed and lactic acid bacteria (LAB) and *B. thermosphacta* predominate, mainly producing organic acids and low-molecular-weight volatiles [2,3,24]. The principal chemical indicators of spoilage include the accumulation of TVB-N, trimethylamine (TMA), and biogenic amines, all resulting from amino acid and NPN degradation [2,3,4,8,24,25,26,27]. According to the European Commission Decision 95/149/EC, TVB-N remains a reliable parameter for determining the acceptability of fishery products at the end of shelf life [28,29]. The reduction in trimethylamine-N-oxide (TMAO)—an osmolyte typical of marine species—is another key of biochemical transformation. TMAO can be converted by bacterial activity, mainly *Pseudomonas* spp. and *Shewanella putrefaciens*, into TMA, dimethylamine (DMA), and monomethylamine and by endogenous enzymes in formaldehyde (FA), which is further oxidized to formic acid (HCOOH) [2,3,9,25,30,31,32,33,34]. The concentration of FA and formic acid thus reflects the extent of TMAO degradation and the advancement of spoilage [30,33,35,36,37,38,39,40,41,42,43,44]. EFSA has confirmed that the majority of formaldehyde detected in fish originates from natural post-mortem biochemical reactions rather than external contamination [37]. During storage, spoilage microorganisms also generate a complex array of volatile organic compounds (VOCs), including aldehydes, ketones, alcohols, and esters, which contribute to species-specific aroma deterioration [2,3,4,24,45,46,47,48]. In addition, *Hafnia alvei*, *Pseudomonas* spp., *Proteus* spp., *Shewanella putrefaciens*, and *Morganella morganii* lead to the accumulation of biogenic amines, such as histamine, putrescine, and cadaverine, which not only contribute to unpleasant odors but also pose toxicological risks when present at high levels of about 200–400 ppm [21,49,50,51,52,53]. Various preservation techniques—refrigeration, vacuum and modified-atmosphere packaging, the use of natural preservatives such as essential oils, and bioprotective cultures— are applied to extend the shelf life and the safety of fresh fish [54,55,56]. The integration of bioprotective cultures with other mild preservation technologies, including sodium alginate coatings or low-temperature MAP, has demonstrated synergistic effects in maintaining the freshness of sea bass and sea bream filets during refrigerated storage [1,15,55,57,58]. However, although these strategies slow microbial proliferation, they do not fully prevent spoilage or guarantee consistent quality over time [1,12,15,29,57,58,59,60,61,62,63]. Considering the combined biochemical, microbiological, and physicochemical processes underlying fish spoilage, the present study aims to establish acceptability limits for both sea bass and sea bream stored under vacuum (VP) and modified-atmosphere (MAP) conditions at 4 ± 2 °C. The evaluation is based on the quantitative assessment of total volatile basic nitrogen (TVB-N), formaldehyde, and formic acid concentrations as objective indicators of freshness loss and product rejection thresholds.

## 2. Materials and Methods

### 2.1. Samples and Packaging

Three lots of farmed, gutted sea bass (*Dicentrarchus labrax*; 578–635 g; ~35 cm) and sea bream (*Sparus aurata*; 440–530 g; ~25 cm) were obtained from Azienda Ittica Valle Dell’Ovo (Str. Oltregorgo 11, 33050 Carlino, Udine, Italy). Fish were reared in sea cages, harvested, and slaughtered after a water–ice bath. Post-mortem holding was 24 h on ice before VP or MAP. Packaging was performed with an Orved VM53 machine (Orved, Musile di Piave, Italy). VP was set to −1.0 bar; MAP used 70% N_2_, <1% O_2_, and 30% CO_2_. The multilayer film consisted of PET (12 µm), aluminum (9 µm), nylon (15 µm), and PE (75 µm). Samples were stored at 4 ± 2 °C, and the temperature was checked daily by a certified thermometer (Tecnafood, Bomporto, Modena, Italy). On days 0, 6, 9, 12, and 15, three different fish samples per treatment were collected for microbiological and physicochemical analyses (pH, TBARS, TVB-N, formaldehyde, and formic acid). Additional samples underwent sensory testing.

### 2.2. Microbiological Analyses

Microbiological analysis were performed as follows: (i) total viable counts (TVCs) were determined on Plate Count Agar (Oxoid, Milan, Italy) at 30 °C for 48–72 h; (ii) LAB were enumerated on MRS agar (Oxoid, Milan, Italy) at 30 °C for 48 h, (iii) total coliforms were counted on VRBLA (Oxoid, Milan, Italy) at 37 °C for 24–48 h; (vi) Enterobacteriaceae on VRBGA (Oxoid, Milan, Italy) at 37 °C for 48 h; (v) *E. coli* on Coli-ID (bioMérieux, Marcy l’Etoile, France) at 37 °C for 48 h; (vi) *Pseudomonas* spp. on *Pseudomonas* Agar Base with CFC (Oxoid, Milan, Italy) at 30 °C for 48 h; (vii) Enterococci on Kanamycin Aesculin agar (Oxoid, Milan, Italy) at 37 °C for 48 h and (viii) sulfite-reducing clostridia were quantified in DRCM (VWR, Radnor, PA, USA) at 37 °C for 24–48 h under anaerobiosis (BBL gas-pack system; Becton Dickinson, Franklin Lakjes, NJ, USA). *Salmonella* spp. and *Listeria monocytogenes* were investigated according to ISO 6579-1 and ISO 11290-1, respectively [64,65].

### 2.3. Physico-Chemical Analyses

The pH value was measured using a pH meter (Basic 20; Crison Instruments, Barcelona, Spain), by inserting the pH meter probe into 3 different points on each sample. The Pearson method [66] was used to evaluate the TVB-N concentration and Ke et al.’s [67] method was used to evaluate the oxidation stability during storage (thiobarbituric acid-reactive substances, TBARS). Formaldehyde and formic acid were determined through the method suggested by Cantoni et al. [33].

### 2.4. Sensory Analysis

Triangle tests [68] were conducted with 20 non-professional assessors (10 women and 10 men; food technology students/consumers). Considering that the assessors were meant to represent the traditional consumers, they did not receive any specific guidance or selection criteria. Cooked samples (wrapped in aluminum foil; 180 °C for 30 min) were presented in randomized order. Samples stored for 0, 12, and 15 days were compared (VP and MAP; both species). Samples collected at the beginning of the storage period (0 days) were compared with those stored for 12 and 15 days, in order to evaluate changes in quality parameters over time. In addition, samples stored for 12 days were compared with those stored for 15 days to assess the progression of spoilage and to determine acceptability limits. In summary:

1. A comparative analysis was conducted on gutted sea bass and sea bream filets packaged under vacuum (VP) conditions and stored at 4 ± 2 °C for 0, 12, and 15 days. Ten samples from each fish species and packaging type were analyzed at each sampling time.
2. A parallel comparison was performed on modified-atmosphere packaged (MAP) samples of the same fish species, stored under the same temperature and time conditions (0, 12, and 15 days). During sensory evaluation, the panelists were presented with two coded samples and asked to identify whether a perceptible difference existed between them. If a difference was detected, assessors were requested to indicate the preferred sample and to express their overall acceptance of the product. The scoring system used was as follows: 1 (excellent), 2 (good), 3 (sufficient), and 4 (scarce).

Assessors identified the odd sample and indicated preference/acceptability. Statistical evaluation followed ISO 4120 and Stone and Sidel [68,69].

### 2.5. Statistical Analysis

Data were analyzed using Statistica software version 7.0 (Statsoft Inc., Tulsa, OK, USA). The values of the different parameters were compared by a one-way analysis of variance, and the means were then compared using Tukey’s honest significance test. Differences were considered significant at *p* < 0.05.

## 3. Results and Discussion

### 3.1. Microbial and Physico-Chemical Characteristic of Sea Bass

At the beginning of storage (day 0), fish freshness was excellent, because they were slaughtered, stored in ice, and used within 24 h; however, as microbial growth and TVB-N values increased, freshness gradually declined over time in both VP and MAP samples. Overall, VP packaging appeared to maintain freshness slightly better than MAP, likely due to a more stable low-oxygen environment limiting the proliferation of aerobic spoilage bacteria (Table 1). Specifically, freshness remained at an excellent level up to day 6, while samples stored for 12 days were still considered acceptable based on microbial load and TVB-N concentrations (Table 1 and Table 2), as suggested by ICMSF [60,70] and Parlapani et al. [2,3,4], respectively. This observation is consistent with the literature indicating that the shelf life of fish depends primarily on storage temperature, atmosphere composition, initial microbial contamination, and handling operations such as gutting, fileting, and packaging [2,3]. Consequently, reported shelf-life durations vary considerably among studies. In general, for fresh whole or gutted sea bass stored in air or MAP and kept on ice or at 4 °C, shelf-life ranges between 8 and 19 days [7,71,72,73]. Our results, however, differ slightly from previous studies [2,3,54,55], which reported shorter shelf-life values—around 8 days for fileted fish stored at 2–4 °C under air and approximately 12 days under MAP conditions.

At day 0, the total viable count (TVC) of *Pseudomonas* spp. and Enterobacteriaceae loads were approximately 2 log CFU/g, while other investigated microorganisms, including *E. coli*, total coliforms, *Clostridium* (H_2_S-producing), lactic acid bacteria (LAB), and Enterococci, were below the detection limits of the analytical methods used. *Listeria monocytogenes* and *Salmonella* spp. were not detected in any sample, confirming the good hygienic quality of the fish. Thiobarbituric acid-reactive substances (TBARS), indicative of lipid oxidation, were initially within acceptable limits (Table 1), as suggested by Che Man and Ramadas [56].

After 15 days of storage, all microbial groups, except *Pseudomonas* spp. and *Clostridium* (H_2_S-producing), showed significant increases. In sea bass, TVC reached approximately 7.8 log CFU/g in VP and 8.5 log CFU/g in MAP samples, surpassing the generally accepted microbial threshold for fish spoilage [60]. Enterobacteriaceae and total coliforms grew at a slower rate than TVC but still reached concentrations above 3–4 log CFU/g by the end of storage. Enterococci exhibited a slight increase up to day 6 (*p* < 0.05), after which the population remained stable until day 15.

Both VP and MAP systems, characterized by low oxygen levels (<0.5%), promoted LAB growth, which reached 5.3–5.6 log CFU/g (Table 1). LAB counts were slightly higher under VP than MAP, whereas total coliform counts were lower under VP. These results align with previous findings showing that oxygen depletion and CO_2_ enrichment inhibit the proliferation of Gram-negative bacteria (e.g., *Pseudomonas* spp.) while favoring Gram-positive microaerophilic LAB [2,3]. Since *Pseudomonas* spp. are strictly aerobic and major spoilers of fish stored in air, their growth was limited under both VP and MAP, remaining nearly constant at 2.1–2.2 log CFU/g through storage (*p* > 0.05). Consequently, the suppression of *Pseudomonas* contributes to prolonged freshness under reduced-oxygen conditions.

The pH and TBARS values did not significantly change (*p* > 0.05) despite the microbial increase (Table 1). TBARS values rose slightly from 1.5 nmol/g at day 0 to 2.4 nmol/g at day 12 and 2.2 nmol/g at day 15, remaining well below rancidity thresholds. The differences through the days were not significative (*p* > 0.05), considering the standard deviations (Table 1), and could be due to the fact that the samples that were investigated at each time were different. According to Pearson [66] and Che Man and Ramadas [56], foods are considered non-rancid when TBARS <8 nmol/g, slightly rancid at 9–20 nmol/g, and rancid/unacceptable when >21 nmol/g. The sensory panel confirmed that no rancid off-flavors were detected, supporting the chemical data.

TVB-N, formaldehyde, and formic acid values of gutted sea bass are reported in Table 2.

All parameters changed significantly during storage (*p* < 0.05), confirming microbial and enzymatic activity as the main drivers of spoilage [2,3,9,23,55,62]. Initial TVB-N was around 13.0 mg N/100 g, increasing progressively to 41.9 mg N/100 g in VP and 43.3 mg N/100 g in MAP by day 15. Spoilage onset was observed around day 12, when TVB-N reached 32.2 mg N/100 g in VP and 33.4 mg N/100 g in MAP—values still below the European Commission limit of 35 mg N/100 g for acceptability [29,74]. Typically, initial TVB-N values range from 5 to 20 mg N/100 g, and concentrations above 30–35 mg N/100 g are generally considered indicative of loss of freshness [9,23,59,60]. Despite the rise in microbial and TVB-N levels, sensory assessment confirmed that the samples remained acceptable at day 12, consistent with the findings of Cakli et al. [9,23,29], who reported similar results for aquacultured sea bass stored in ice with TVC >8 log CFU/g, TVB-N around 50 mg N/100 g, and TBARS near 2.6 nmol/g.

Formaldehyde and formic acid levels also changed significantly in both VP and MAP samples (*p* < 0.05). These compounds are derived from the degradation of trimethylamine-N-oxide (TMAO) by the endogenous enzyme TMAO demethylase (TMAOase), producing dimethylamine (DMA) and formaldehyde (FA), part of which is oxidized to formic acid [32], according to the formula: “2HCHO + O_2_ > 2 HCOOH”. In this study, initial FA concentrations were 3.9 mg/kg in VP and 4.5 mg/kg in MAP, peaking at day 6 (28.4 mg/kg and 32.7 mg/kg, respectively), before decreasing as conversion to formic acid occurred. Formic acid levels, conversely, increased steadily through storage—from 1.8 mg/kg to 5.4 mg/kg in VP and from 2.9 mg/kg to 5.3 mg/kg in MAP. These values are higher than those reported by previous studies [33,35] for mullet, where the levels were about 1.4–4.8 mg/kg, and largely lower for cod and deep-frozen hake, presenting levels between 100 mg/kg and 232–293 mg/kg, respectively [36,38,39,40,41,42,43,44]. It was observed that there was a high variability for formic acid in MAP samples (*p* < 0.05). It can be hypothesized that this variability is due to the sample, which varied over time, and to a higher concentration of residual oxygen in the packaging, increasing the oxidation of formaldehyde.

Because formaldehyde may accumulate naturally through post-mortem degradation, its intentional addition to fresh fish is illegal and poses health risks [35,37,75].

Furthermore, considering all the parameters investigated, no differences were observed between the samples stored in VP and in MAP (*p* > 0.05).

As shown, it is possible to conclude that the sea bass samples can be largely accepted at 12 days of storage.

### 3.2. Microbial and Physico-Chemical Characteristic of Sea Bream

In the case of sea bream (Table 3), all microbial groups except *Clostridium* (H_2_S-producing) and Enterococci increased during storage.

Initial TVC was 2.1 log CFU/g, reflecting high initial quality and good hygienic handling during farming and packaging [2,3,53,54]. At 12 days, TVC reached 5.5 ± 0.1 log CFU/g in VP and 5.7 ± 0.3 log CFU/g in MAP. These results agree with previous observations for gutted and ungutted sea bream stored at 4 °C [9,23], confirming 7 log CFU/g as a reasonable limit for acceptability [60]. However, at 15 days TVC reached approximately 7.9 log CFU/g in VP and 8.2 log CFU/g in MAP samples and, consequently, exceed the generally accepted microbial threshold for fish spoilage [60].

Enterobacteriaceae increased from ~2 log CFU/g to 4.2–4.4 log CFU/g, and total coliforms reached 3.6–3.8 log CFU/g at 12 days. *E. coli* and Enterococci showed slight increases up to day 6, stabilizing thereafter, possibly due to their mesophilic nature.

*Pseudomonas* spp. did not show significant changes (*p* > 0.05), likely because of residual low oxygen in both packaging systems, confirming that these aerobic species are not dominant spoilers under VP or MAP for Mediterranean fish [7,34,46,47,48,76,77]. The most abundant microbial group was LAB, which are microaerophilic and well-adapted to low-O_2_ environments [2,3,76,77]. LAB reached 5.7 ± 0.1 log CFU/g in VP and 5.5 ± 0.2 log CFU/g in MAP, confirming that these conditions favor their proliferation. *L. monocytogenes* and *Salmonella* spp. were absent from all samples, demonstrating good hygienic practices through processing.

The pH and TBARS values of sea bream also remained stable (*p* > 0.05). TBARS increased slightly up to day 6 (*p* < 0.05) and then remained constant, reaching final values of 2.4 and 2.3 nmol/g in VP and MAP, respectively. Also, for the sea bream samples, there were no rancid off-flavors, considering the low TBARS level, as confirmed by the sensory panel.

Conversely, TVB-N (Table 4) values increased significantly during storage (*p* < 0.05), confirming the effects of the microbial growth, as suggested by different authors [2,3,9,23,34,59,60].

At the beginning of the storage, TVB-N values of the tested gutted sea bream were quite similar to those of sea bass and were 12.9 mg N/100 g in VP and 12.7 mg N/100 g in MAP samples. Then, they increased, and at 15 days storage reached values about 41.5 ± 0.3 mg N/100 g in VP and 42.8 ± 0.8 mg N/100 g (Table 4). These TVB-N concentrations are considered too high by the EEC/1995 [74], which accepts values up to 35 mg N/100 g. Indeed, at 12 days, in both the samples stored either in VP or in MAP, the TVB-N concentrations were always less than 35 mg N/100 g product. For this reason, the level of TVB-N at 12 days must be considered largely acceptable according to the limit proposed by the EEC/1995 [74], which is 35 mg N/100 g. Also, for sea bream samples, the initial TVB-N concentration was between 5 and 20 mg N/100 g [9,23], and at 12 days storage its level was close to 30–35 N/100 g, which is the concentration generally regarded as the limit of acceptability for ice-stored cold water fish [55,60,61]. The final TVB-N concentration in sea bream samples was quite similar to the ones of sea bass samples, despite that the level of TVC and Enterobacteriaceae concentration in sea bream was greater than those in sea bass.

Based on microbial data and TVB-N concentration, the sea bream samples must be accepted at 12 days. Indeed, Cakly et al. [9,23,29] suggested the acceptability of aquacultured sea bream (*Sparus aurata*) stored in ice, when the TVC average value was above 8 log CFU/g, and TVB-N and TBARS (malonaldehyde) were about 50.13 ± 0.25 mg N/100 g and 2.66 5 ± 0.06 mg malonaldehyde/kg, respectively.

As expected for sea bass, the formaldehyde and formic acid values also changed either in VP or MAP sea bream samples (*p* < 0.05). Formaldehyde and formic acid in sea bream followed similar kinetics to sea bass (Table 4). Formaldehyde rose from 3.5 mg/kg (VP) and 4.9 mg/kg (MAP) to peak values of 30.2 and 35.2 mg/kg at day 6, before declining as conversion to formic acid occurred. Formic acid increased from approximately 2.1 to 5.4 mg/kg (VP) and from 3.2 to 7.2 mg/kg (MAP).

Indeed, formic acid increases during all of the storage times, evolving from an initial concentration of approximately 2.1 mg/kg to a final average value of 5.4 mg/kg in VP and from 3.2 mg/kg to 7.2 mg/kg in MAP. Both the values are lower than those observed by different authors, who found indigenous formaldehyde levels between 1 and 293 mg/kg [33,34,35,36,37,38,39]. However, both the formaldehyde and formic acid did not influence the acceptability of the fish, because their concentrations were less than 20 mg/kg [33,36,38,39].

As shown for sea bass, based on observations of all the investigated parameters, it is possible to conclude that the sea bream samples can also be largely accepted at 12 days of storage [39,40,41,42,43,44].

Finally, regarding the type of packaging, no differences were observed in all the valued parameters between all the VP and MAP samples.

### 3.3. Sensory Analysis

The sensory characteristics cannot be neglected because they represent the main factors responsible for product acceptance [74]. Indeed, the samples used in this study were also evaluated by non-trained, non-professional assessors. For these reasons, it was decided to show only the overall quality attributes, since the other sensory descriptors recorded a similar trend. Among the non-professional subjects, all the assessors were able to distinguish the different samples by smell, a result that was statistically significant. The sea bass and sea bream samples stored for 15 days were considered unacceptable when they were compared either with the ones of 0 days or 12 days. All the assessors distinguished the difference between the tested samples and recognized that the samples stored until 15 days were spoiled (*p* < 0.05), and their score was scarce. Indeed, according to the triangle test, the minimum number of correct responses needed to establish significance at *p* < 0.05 with 20 assessors is 11 [68,69] and in this case 20 out 20 assessors identified differences. It should be noted that, even when the assessors were unable to discern differences between the samples, the test required them to provide a response. In each case, 7 out 20 non-professional assessors recognized differences when evaluating sea bass and 9 out 20 recognized differences when evaluating sea bream samples (*p* > 0.05). Despite their recognition of the different samples, all the tested samples were considered acceptable. Similarly, the 13 assessors who did not correctly identify the different samples also rated all samples as acceptable. However, the samples at 0 days were scored as excellent and the ones at 12 days as both good (Table 5) and acceptable.

## 4. Conclusions

To propose an objective parameter to define the acceptability of fresh sea bass and sea bream, the evolution of the concentration of formaldehyde, formic acid, and TVB-N was evaluated. As indicated, TVB-N appeared to be the most appropriate index. The formaldehyde value cannot be used because it increased up to 6 days and then decreased because it was transformed into formic acid. Nevertheless, formic acid also cannot be considered a valid parameter, because it reached high values when the sensorial analysis indicated the loss of acceptability. For this reason, TVB-N represents the only parameter of interest for the purpose of defining the acceptability of both the types of the investigated fresh fish and it can be accepted as the freshness index. Considering the results of the microbial, physico-chemical, and sensory analyses, a level of TVB-N less than or equal to 35 mg N/100 g of product was proposed as the acceptability limit for sea bass and sea bream, stored either in VP or in MAP at 4 ± 2 °C. Consequently, for both fish species, a shelf-life limit of 12 days was also suggested.

## Figures and Tables

**Table 1:** Fate of microorganisms, pH, and TBARS in gutted sea bass packaged under vacuum or in MAP and stored at 4 ± 2 °C.

Parameter	Time (Days)							
	T0		T6		T12		T15	
	VP	MAP	VP	MAP	VP	MAP	VP	MAP
Total viable count	2.0 ± 0.1 ^a^	2.0 ± 0.2 ^a^	5.2 ± 0.3 ^b^	7.7 ± 0.3 ^c^	7.8 ± 0.3 ^c^	8.4 ± 0.2 ^d^	7.5 ± 0.2 ^c^	8.5 ± 0.1 ^d^
Enterobacteriaceae	2.0 ± 0.2 ^a^	2.1 ± 0.3 ^a^	3.3 ± 0.3 ^b^	3.5 ± 0.1 ^b^	4.2± 0.4 ^c^	4.4 ± 0.4 ^c^	4.1 ± 0.3 ^c^	4.2 ± 0.2 ^c^
*Pseudomonas* spp.	2.1 ± 0.3 ^a^	2.2 ± 0.2 ^a^	2.1 ± 0.3 ^a^	2.2 ± 0.1 ^a^	2.2 ± 0.3 ^a^	2.0 ± 0.3 ^a^	2.1 ± 0.2 ^a^	2.2 ± 0.1 ^a^
*E. coli*	<10 ^a^	<10 ^a^	2.0 ± 0.2 ^b^	2.0 ± 0.1 ^b^	2.0 ± 0.1 ^b^	2.0 ± 0.2 ^b^	2.1 ± 0.1 ^b^	2.1 ± 0.2 ^b^
Total coliforms	<10 ^a^	<10 ^a^	2.2 ± 0.2 ^b^	2.4 ± 0.1 ^b^	4.1 ± 0.2 ^c^	4.5 ± 0.2 ^c^	3.9 ± 0.5 ^c^	4.7 ± 0.3 ^c^
*Clostridium* H_2_ S+	<10 ^a^	<10 ^a^	<10 ^a^	<10 ^a^	<10 ^a^	<10 ^a^	<10 ^a^	<10 ^a^
Lactic acid bacteria	<10 ^a^	<10 ^a^	2.2 ± 0.1 ^a^	2.3 ± 0.1 ^a^	5.5 ± 0.2 ^a^	5.2 ± 0.3 ^b^	5.6 ± 0.2 ^b^	5.3 ± 0.1 ^b^
Enterococci	<10 ^a^	<10 ^a^	2.0 ± 0.1 ^a^	2.1 ± 0.1 ^a^	2.1 ± 0.2 ^a^	2.2 ± 0.2 ^a^	2.1 ± 0.2 ^a^	2.1 ± 0.1 ^a^
TBARS	1.5 ± 0.2 ^a^	1.5 ± 0.1 ^a^	2.3± 0.2 ^b^	2.3 ± 0.2 ^b^	2.4 ± 0.2 ^b^	2.3 ± 0.1 ^b^	2.2 ± 0.3 ^b^	2.2 ± 0.2 ^b^
pH	6.12 ± 0.03 ^a^	6.10 ± 0.02 ^a^	6.09 ± 0.05 ^a^	6.01 ± 0.04 ^a^	6.05 ± 0.04 ^a^	6.02 ± 0.03 ^a^	6.03 ± 0.04 ^a^	6.05 ± 0.02 ^a^

VP: vacuum packaging; MAP: modified-atmosphere packaging; data represent the means ± standard deviations of the total samples; mean with the same letters within a lane (following the values), considering each single parameter of MAP, VP, and time are not significantly different (*p* < 0.05). Analyses were conducted in triplicate on three different samples per each sampling point. Data log CFU/g; < 10 CFU/g, lower limit of the microbial method; TBARS: nmol malonaldheyde/g.

**Table 2:** Physico-chemical evolution in gutted sea bass packaged in VP or in MAP and stored at 4 ± 2 °C.

Days	Sea Bass					
	VP			MAP (20% CO_2_; 80% N_2_; < 0.5% O_2_)		
	TVB-N	Formaldehyde	Formic Acid	TVB-N	Formaldehyde	Formic Acid
**0**	13.0 ± 0.2 ^a^	3.9 ± 0.1 ^a^	1.8 ± 0.3 ^a^	13.5 ± 0.5 ^a^	4.5 ± 0.3 ^a^	2.9 ± 0.3 ^a^
**6**	19.9 ± 0.5 ^a^	28.4 ± 0.4 ^b^	3.6 ± 0.2 ^b^	20.1 ± 0.3 ^a^	32.7 ± 0.3 ^b^	4.4 ± 0.2 ^b^
**9**	31.5 ± 1.3 ^a^	12.5 ± 0.2 ^c^	5.2 ± 0.1 ^c^	33.2 ± 0.3 ^a^	12.5 ± 0.5 ^c^	5.8 ± 0.1 ^c^
**12**	32.2 ± 0.9 ^a^	14.5 ± 0.7 ^d^	5.5 ± 0.2 ^d^	33.4 ± 0.7 ^a^	15.5 ± 0.2 ^d^	6.1 ± 0.3 ^c^
**15**	41.9 ± 0.2 ^b^	14.5 ± 0.1 ^d^	5.4 ± 0.1 ^d^	43.3 ± 0.2 ^b^	14.5 ± 0.7 ^d^	5.3 ± 0.2 ^d^

VP: vacuum packaging; MAP: modified-atmosphere packaging; data represent means ± standard deviations of the total samples; mean with the same letters within the columns (following the values) considering each single parameter of MAP, VP, and time are not significantly different (*p* < 0.05). TVB-N (total volatile basic nitrogen), mg N/100; formaldehyde and formic acid, mg/kg.

**Table 3:** Fate of microorganisms, pH, and TBARS in gutted sea bream packaged under vacuum or in MAP and stored at 4 ± 2 °C.

Parameter	Time (Days)							
	T0		T6		T12		T15	
	VP	MAP	VP	MAP	VP	MAP	VP	MAP
Total viable count	2.1 ± 0.2 ^a^	2.1 ± 0.1 ^a^	4.4 ± 0.5 ^b^	5.3 ± 0.3 ^b^	5.5 ± 0.1 ^b^	5.7 ± 0.3 ^b^	7.9 ± 0.2 ^c^	8.2 ± 0.1 ^c^
Enterobacteriaceae	2.1 ± 0.1 ^a^	2.0 ± 0.2 ^a^	2.2 ± 0.3 ^a^	2.2 ± 0.2 ^a^	4.2 ± 0.2 ^b^	4.4 ± 0.2 ^b^	4.6 ± 0.2 ^b^	4.6 ± 0.3 ^b^
*Pseudomonas* spp.	2.2 ± 0.1 ^a^	2.1 ± 0.2 ^a^	2.1 ± 0.1 ^a^	2.2 ± 0.2 ^a^	2.2 ± 0.1 ^a^	2.2 ± 0.2 ^a^	2.3 ± 0.2 ^a^	2.3 ± 0.2 ^a^
*E. coli*	<10 ^a^	<10 ^a^	1.4 ± 0.2 ^a^	1.5 ± 0.1 ^a^	1.5 ± 0.1 ^a^	1.4 ± 0.1 ^a^	1.6 ± 0.2 ^a^	1.6 ± 0.2 ^a^
Total coliforms	<10 ^a^	<10 ^a^	2.0 ± 0.3 ^b^	2.2 ± 0.4 ^b^	3.6 ± 0.2 ^b^	3.8 ± 0.3 ^b^	4.4 ± 0.3 ^c^	4.4 ± 0.1 ^c^
*Clostridium* H_2_S+	<10	<10	<10	<10	<10	<10	<10	<10
Lactic acid bacteria	<10 ^a^	<10 ^a^	2.2 ± 0.4 ^a^	2.2 ± 0.2 ^a^	5.8 ± 0.2 ^a^	4.9 ± 0.5 ^b^	5.7 ± 0.1 ^a^	5.5 ± 0.2 b
Enterococci	<10 ^a^	<10 ^a^	1.5 ± 0.1 ^b^	1.6 ± 0.1 ^b^	1.5 ± 0.1 ^b^	1.6 ± 0.1 ^a^	1.6 ± 0.2 ^b^	1.7. ± 0.2 ^b^
TBARS	1.5 ± 0.1 ^a^	1.7 ± 0.2 ^a^	2.3 ± 0.2 ^b^	2.4 ± 0.2 ^b^	2.4 ± 0.1 ^b^	2.5 ± 0.1 ^b^	2.4 ± 0.1 ^b^	2.3 ± 0.1 ^b^
pH	6.10 ± 0.01 ^a^	6.00 ± 0.02 ^a^	6.09 ± 0.03 ^a^	6.09 ± 0.02 ^a^	5.91 ± 0.02 ^a^	6.03 ± 0.01 ^a^	6.02 ± 0.02 ^a^	6.03 ± 0.02 ^a^

VP: vacuum packaging; MAP: modified-atmosphere packaging; data represent the means ± standard deviations of the total samples; mean with the same letters within a lane (following the values), considering each single parameter of MAP, VP, and time are not significantly different (*p* < 0.05). Analyses were conducted in triplicate on three different samples per each sampling point. Data log CFU/g; < 10 CFU/g, lower limit of the microbial method; TBARS: nmol malonaldheyde/g.

**Table 4:** Physico-chemical evolution in gutted sea bream packaged in VP or in MAP and stored at 4 ± 2 °C.

Days	Sea Bream					
	VP			MAP (20% CO_2_; 80% N_2_; < 0.5% O_2_)		
	TVB-N	Formaldehyde	Formic Acid	TVB-N	Formaldehyde	Formic Acid
**0**	12.9 ± 0.5 ^a^	3.5 ± 0.1 ^a^	2.1 ± 0.3 ^a^	12.7 ± 0.3 ^a^	4.9 ± 0.4 ^a^	3.2 ± 0.1 ^a^
**6**	21.9 ± 0.1 ^a^	30.2 ± 0.2 ^b^	4.2 ± 0.3 ^b^	23.5 ± 1.2 ^a^	35.2 ± 0.1 ^b^	6.1 ± 0.2 ^b^
**9**	31.5 ± 1.3 ^a^	12.5 ± 0.1 ^c^	5.3 ± 0.2 ^c^	33.2 ± 0.3 ^a^	12.5 ± 0.3 ^c^	5.8 ± 0.3 ^b^
**12**	34.6 ± 0.2 ^a^	12.7 ± 0.2 ^c^	5.4 ± 0.3 ^c^	34.0 ± 0.5 ^a^	17.3 ± 0.2 ^d^	7.2 ± 0.4 ^c^
**15**	41.5 ± 0.3 ^b^	12.3 ± 0.3 ^c^	5.4 ± 0.2 ^c^	42.8 ± 0.8 ^b^	17.4 ± 0.1 ^d^	7.2 ± 0.2 ^c^

VP: vacuum packaging; MAP: modified-atmosphere packaging; data represent the means ± standard deviations of the total samples; mean with the same letters within the columns (following the values), considering each single parameter, MAP, VP, and time are not significantly different (*p* < 0.05). TVB-N (total volatile basic nitrogen), mg N/100; formaldehyde and formic acid: mg/kg.

**Table 5:** Sensory evaluation by not professionally trained panelists.

Fish	Samples	Difference	Final Values Scores *
**Sea bass**	0 days vs. 12 days	+7/20	1/2 a/a
	0 days vs.15 days	+20/20	1/4 a/na
	12 days vs. 15 days	+20/20	2/4 a/na
**Sea bream**	0 days vs. 12 days	+9/20	1/2 a/a
	0 days vs. 15 days	+20/20	1/4 a/na
	12 days vs. 15 days	+20/20	2/4 a/na

Legend: + n. positive assessments/total assessments. * Scores (samples vs. samples): 1 (excellent); 2 (good); 3 (sufficient); 4 (scarce); a: acceptable; na: non-acceptable.

## Data Availability

The original contributions presented in this study are included in the article. Further inquiries can be directed to the corresponding author.
